# Publisher Correction: Co-targeting CDK4/6 and AKT with endocrine therapy prevents progression in CDK4/6 inhibitor and endocrine therapy-resistant breast cancer

**DOI:** 10.1038/s41467-021-25901-z

**Published:** 2021-09-16

**Authors:** Carla L. Alves, Sidse Ehmsen, Mikkel G. Terp, Neil Portman, Martina Tuttolomondo, Odd L. Gammelgaard, Monique F. Hundebøl, Kamila Kaminska, Lene E. Johansen, Martin Bak, Gabriella Honeth, Ana Bosch, Elgene Lim, Henrik J. Ditzel

**Affiliations:** 1 Department of Cancer and Inflammation Research, Institute of Molecular Medicine, University of Southern Denmark, Odense, Denmark; 2 Department of Oncology, Institute of Clinical Research, Odense University Hospital, Odense, Denmark; 3 Garvan Institute of Medical Research, Sydney, NSW Australia; 4 St. Vincent’s Clinical School, Faculty of Medicine, University of New South Wales Sydney, Sydney, NSW Australia; 5 Division of Oncology and Pathology, Department of Clinical Sciences, Lund University, Lund, Sweden; 6 Department of Pathology, Sydvestjysk Sygehus, Esbjerg, Denmark; 7 Academy of Geriatric Cancer Research (AgeCare), Odense University Hospital, Odense, Denmark

**Keywords:** Translational research, Breast cancer

Correction to: *Nature Communications* 10.1038/s41467-021-25422-9, published online 25 August 2021.

The original HTML version of this Article was updated shortly after publication because the previous HTML version linked to an incorrect Transparent Peer Review file.

## Supplementary information

Updated Peer Review File

