# Supplementary material for: Co-targeting CDK4/6 and AKT with endocrine therapy prevents progression in CDK4/6 inhibitor and endocrine therapy-resistant breast cancer
Source: Nat Commun. 2021 Aug 25;12:5112. doi: 10.1038/s41467-021-25422-9 (PMC8387387; doi:10.1038/s41467-021-25422-9)
Supplement: Supplementary file 1 — Supplementary Information [file 41467_2021_25422_MOESM1_ESM.pdf]

Supplementary Information

Co-targeting CDK4/6 and AKT with endocrine therapy prevents progression in CDK4/6 inhibitor and endocrine therapy-resistant breast cancer

Carla L. Alves, Sidse Ehmsen, Mikkel G. Terp, Neil Portman, Martina Tuttolomondo, Odd L. Gammelgaard, Monique F. Hundebøl, Kamila Kaminska, Lene E. Johansen, Martin Bak, Gabriella Honeth, Ana Bosch, Elgene Lim, Henrik J. Ditzel

Supplementary Figure S1

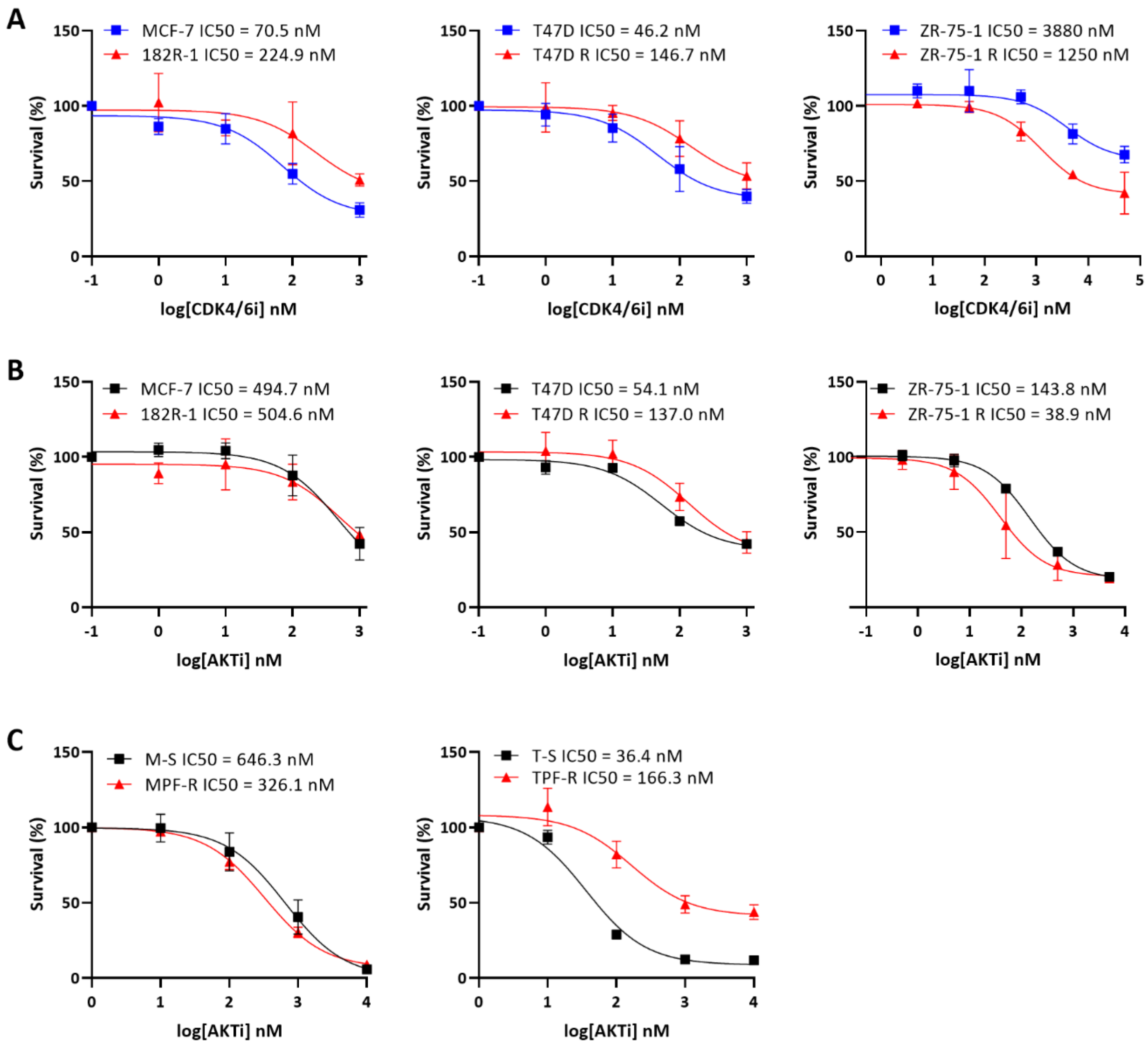

Fig. S1. Dose-dependent curves of CDK4/6 inhibitor and AKT inhibitor in ER+ breast cancer cell models. MCF-7, 182R-1, T47D, T47D R, ZR-75-1 and ZR-75-1 R cells were treated with different concentrations of the CDK4/6 inhibitor (CDK4/6i) palbociclib (**A**) or AKT inhibitor (AKTi) capivasertib (**B**) for 4-6 days. (**C**) M-S, MPF-R, T-S and TPF-R cells were treated with different concentrations of AKTi capivasertib for 4-6 days. Cell growth was measured by crystal violet colorimetric assay. Growth relative to vehicle (%) is plotted against log drug concentration. Each data point represents the mean ± SEM of three technical replicates.

Supplementary Figure S2

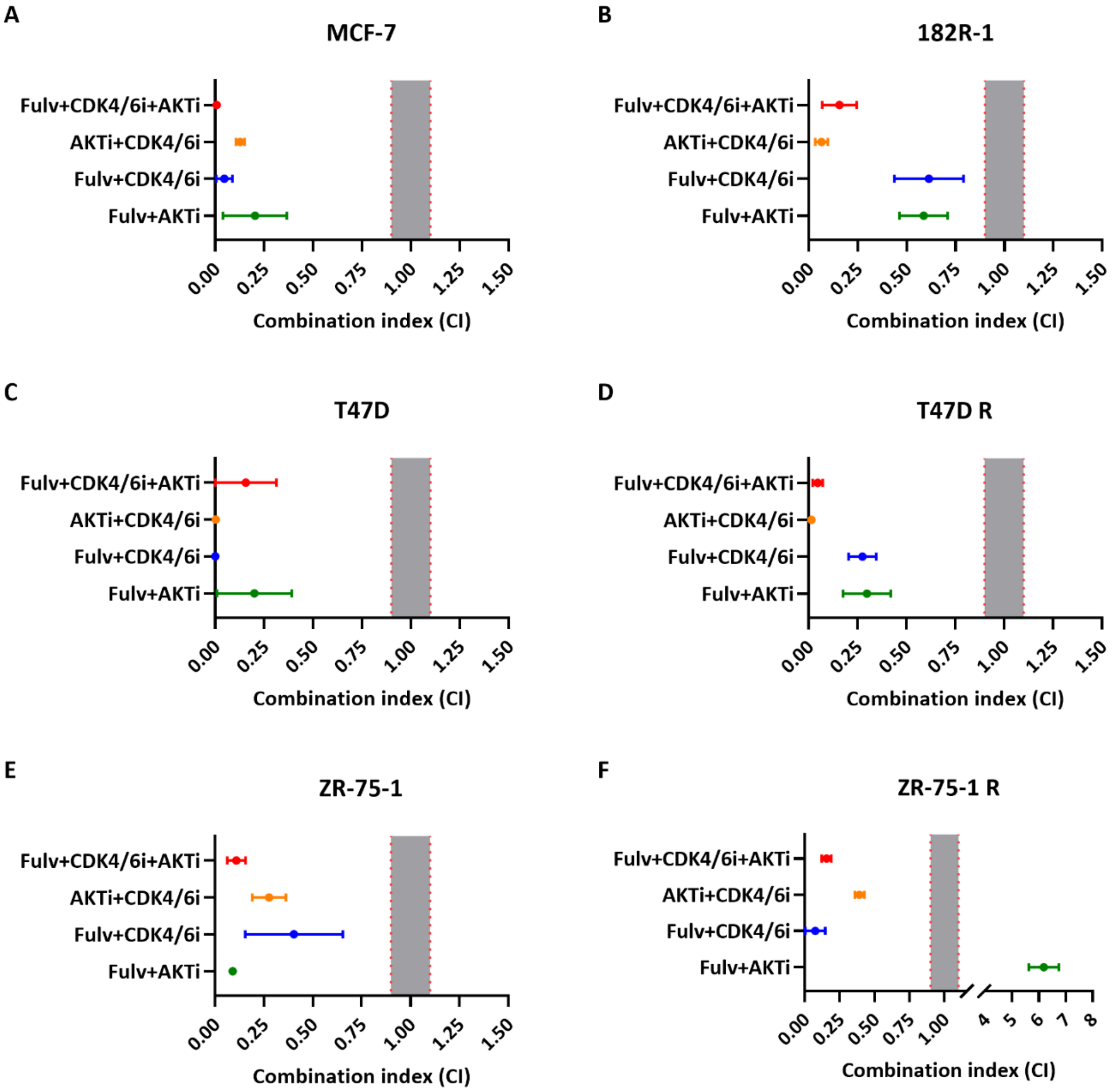

Fig. S2. Combined fulvestrant, palbociclib and capivasertib exhibit synergistic activity in ER+ breast cancer cell lines. Drug interaction analysis between fulvestrant (fulv), CDK4/6 inhibitor (CDK4/6i) palbociclib and AKT inhibitor (AKTi) capivasertib in MCF-7 (**A**), 182R-1 (**B**), T47D (**C**), T47D R (**D**), ZR-75-1 (**E**) and ZR-75-1 R (**F**) breast cancer cell lines. Effect of the drug combination was determined with crystal violet-based colorimetric assay using the combination index (CI) equation from Chou-Talalay method. The gray band indicates the area corresponding to additive interaction. Each data point represents the mean ± SEM of three technical replicates.

Supplementary Figure S3

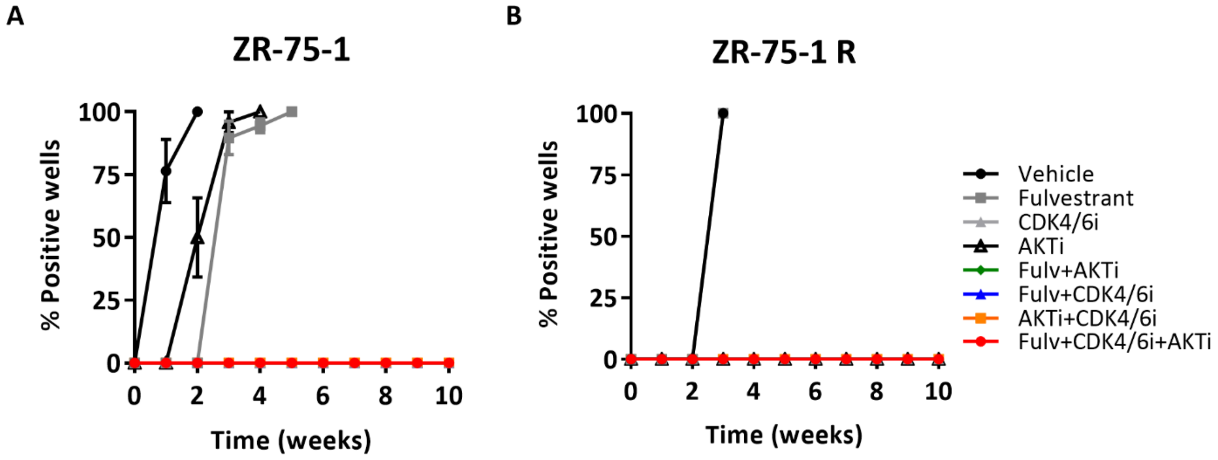

Fig. S3. Evaluation of the outgrowth of resistant colonies in ZR-75-1 breast cancer cell lines treated with fulvestrant, CDK4/6i and AKTi. Outgrowth of resistant colonies was investigated in ZR-75-1 (**A**) and ZR-75-1 R (**B**) cells by weekly evaluation of the percentage of 48 wells at 50% or greater confluence (positive wells) over 10 weeks. Experiments were conducted in three biological replicates and data are shown as mean ± SEM.

Supplementary Figure S4

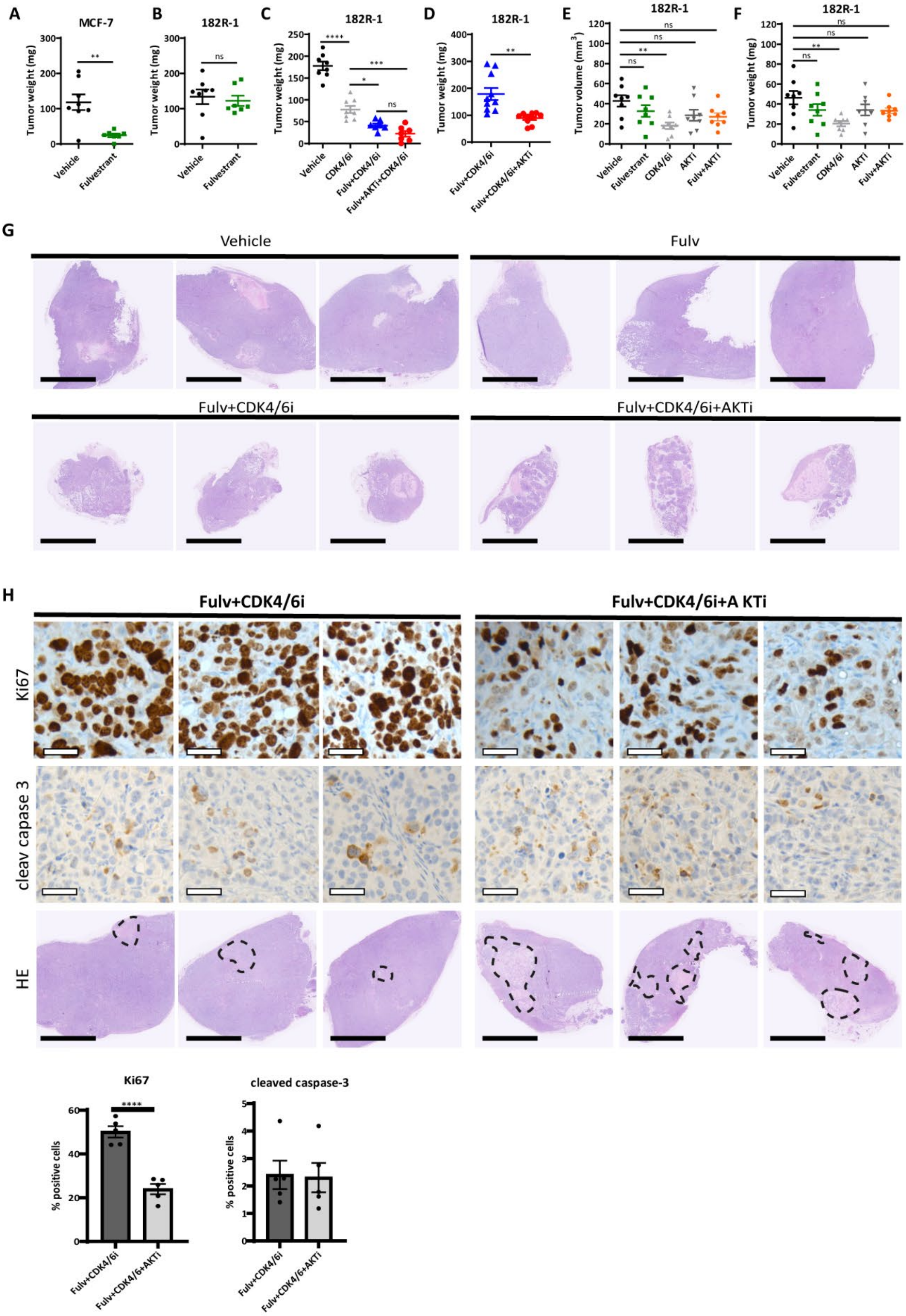

Fig. S4. Combined fulvestrant, CDK4/6i and AKTi prevents progression in tumor xenografts resistant to fulvestrant. Evaluation of MCF-7 (**A**) and 182R-1 (**B**) tumors weight at endpoint (5 weeks) after treatment with fulvestrant (Fulv, 100 mg/Kg bodyweight; once a week subcutaneously; $N = 7$) or vehicle (castor oil; once a week subcutaneously, $N = 8$). Effect on endpoint tumor weight of CDK4/6 inhibitor (CDK4/6i) palbociclib (50 mg/Kg bodyweight; $N = 8$) alone (**C**), in double combination with fulvestrant (100mg/Kg bodyweight; $N = 7$ and $N = 10$) (**C** and **D**), in triple combination with AKT inhibitor (AKTi) capivasertib (100mg/Kg bodyweight; $N = 6$ and $N = 10$) (**C** and **D**), or vehicle (castor oil and 25% w/v HPB cyclodextrin; $N = 8$) (**C**). CDK4/6i and AKTi were administered by oral gavage once daily for 5 days a week, for 6 (**C**) or 8 (**D**) weeks. Evaluation of 182R-1 tumor volume (**E**) and weight (**F**) following 2 weeks of treatment with single agents fulvestrant (100mg/Kg bodyweight; $N = 8$), CDK4/6i (50 mg/Kg bodyweight; $N = 8$) or AKTi (100 mg/Kg bodyweight; $N = 8$), fulvestrant and AKTi combination ($N = 8$) or vehicle (castor oil and 25% w/v HPB cyclodextrin; $N = 8$). Data are shown as mean endpoint tumor weight ± SEM. Asterisks indicate significant difference in ANOVA one-way tests (**C**, **E** and **F**) or two-tailed t-tests (**A**, **B** and **D**) at the endpoint ($p < 0.05$). (**G**) Representative micrographs of the morphology of 182R-1 treated for 6 weeks with vehicle, fulvestrant, combined fulvestrant and CDK4/6i and triple combination with AKTi (3 mice of each treatment group) by HE staining (scale bars, 2.5 mm) (**H**) Representative micrographs of immunohistochemical stainings of 182R-1 tumor sections from 3 mice of each treatment group in **C** showing Ki67 and cleaved caspase-3 expression and morphology by HE staining (scale bars, white: 100 µM, black: 2.5 mm). Quantification of Ki67 and cleaved caspase-3 was performed by Image. Data are shown as mean ± SEM. Asterisks indicate significant differences by one-tailed t-test ($p < 0.05$).

Supplementary Fig. S5

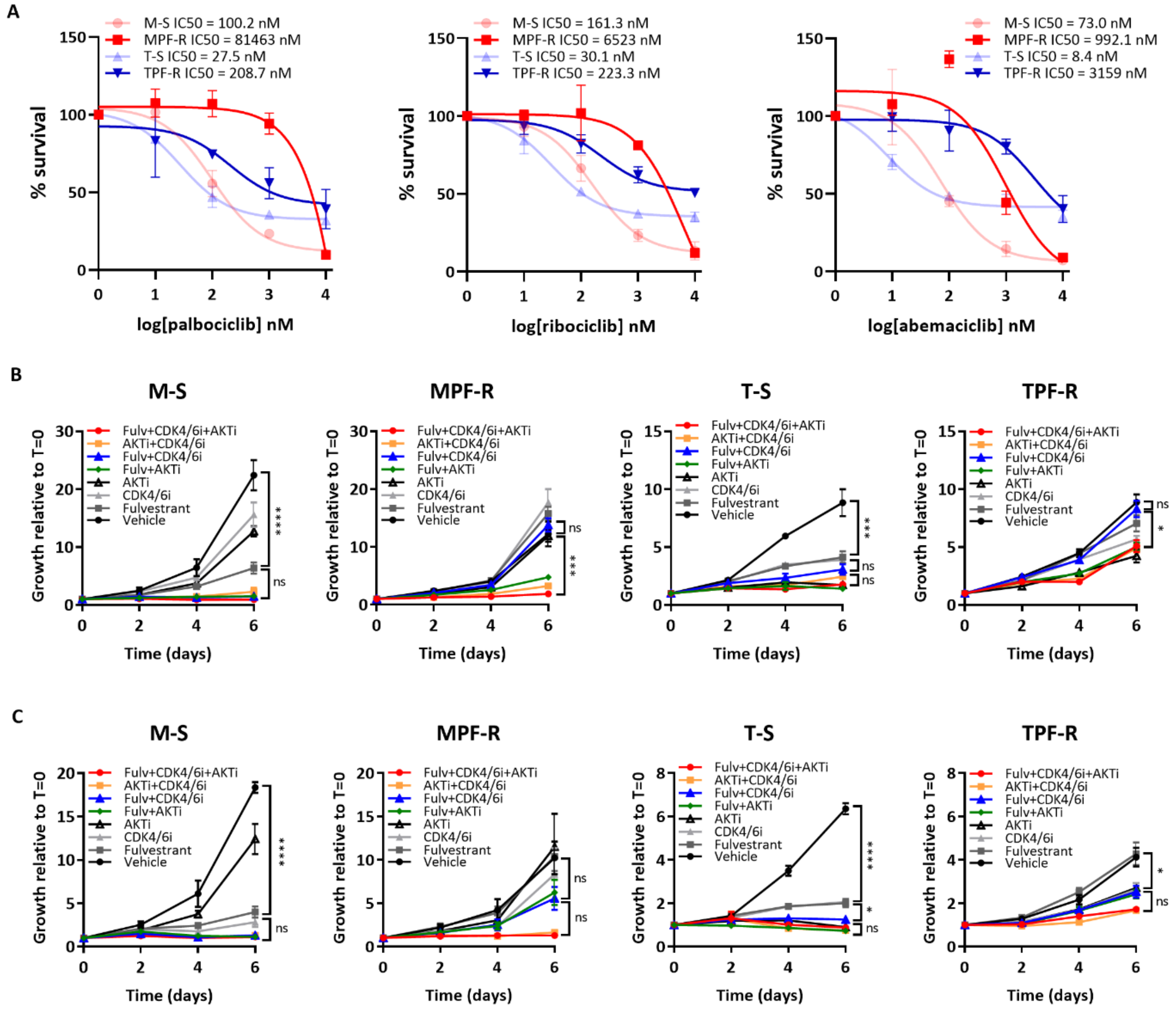

Fig. S5. Triple combination with fulvestrant, AKTi and different CDK4/6i is more effective than standard combined fulvestrant and CDK4/6i in breast cancer cell lines resistant to combined palbociclib and fulvestrant. MPF-R and TPF-R cells were treated with different concentrations of the CDK4/6 inhibitor (CDK4/6i) palbociclib, ribociclib or abemaciclib (**A**) for 6 days. Cell growth was measured by crystal violet colorimetric assay. Growth relative to vehicle (%) is plotted against log drug concentration. Each data point represents the mean ± SEM of three technical replicates. The effect of fulvestrant (Fulv, 100 nM), AKT inhibitor (AKTi) capivasertib

(250 nM in the MCF-7 cell model; 100 nM in the T47D cell model), and CDK4/6 inhibitor (CDK4/6i) ribociclib (200 nM, **B**) or abemaciclib (200 nM, **C**) as single agents or in double and triple combination, was assessed in M-S, MPF-R, T-S and TPF-R cells by crystal violet growth assay performed over 6 days. Growth experiments were conducted in three biological replicates and data are shown as mean ± SEM. Asterisks indicate significant differences in one-way ANOVA tests at day 6 ($p < 0.05$).

Supplementary Figure S6

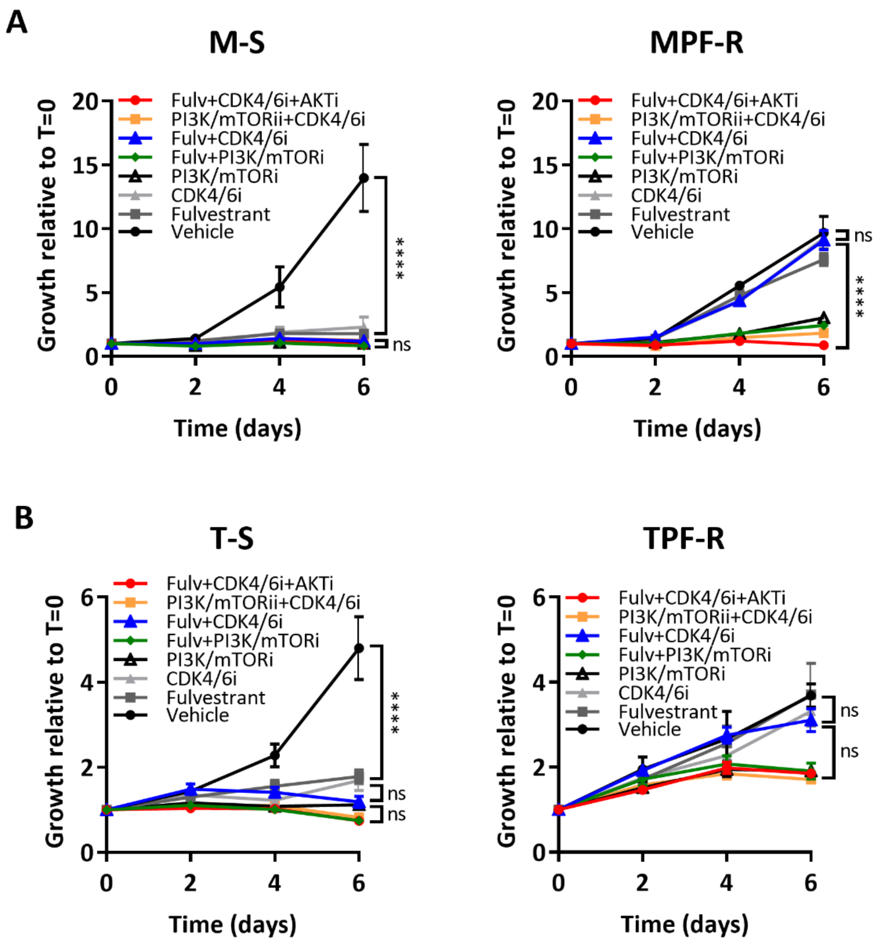

Fig. S6. Triple combination with fulvestrant, CDK4/6i and the dual PI3K/mTORi is more effective than standard combined fulvestrant and CDK4/6i in breast cancer cell lines resistant to combined palbociclib and fulvestrant. The efficacy of fulvestrant (Fulv, 100 nM), CDK4/6 inhibitor (CDK4/6i) palbociclib (200 nM) and the dual PI3K/mTOR inhibitor (PI3K/mTORi) gedatolisib (10 nM) as single agents or in double and triple combination, was assessed in M-S and MPF-R (**A**) and T-S and TPF-R (**B**) cells by crystal violet growth assay performed over 6 days. Growth experiments were conducted in three biological replicates and data are shown as mean ± SEM. Asterisks indicate significant differences in one-way ANOVA tests at day 6 ($p < 0.05$).

Supplementary Fig. S7

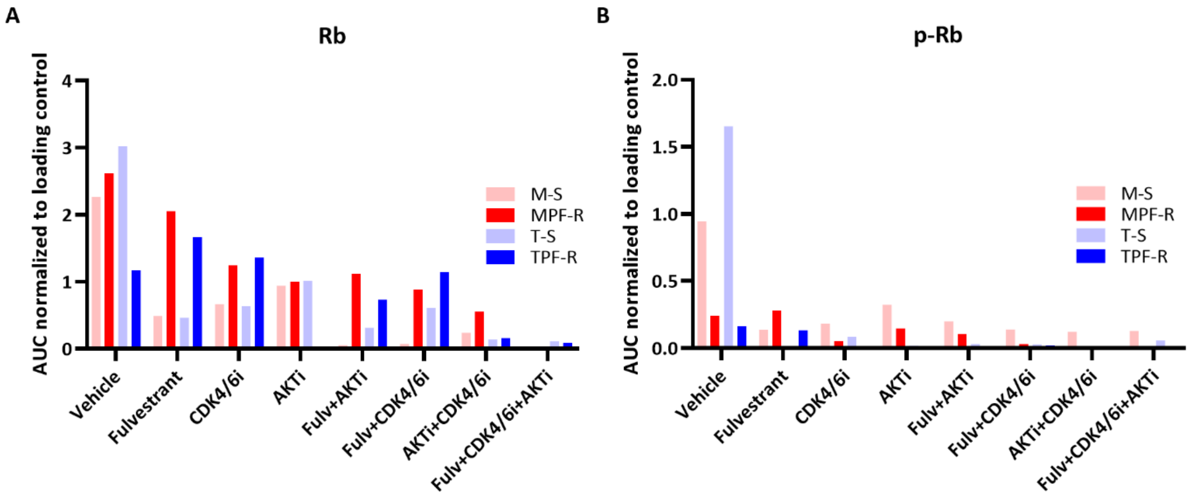

Fig. S7. Breast cancer cell lines resistant to combined CDK4/6i and fulvestrant show reduced dependence on cyclin D1-CDK4/6 pathway. Densitometry analysis of Western blot bands of Rb and p-Rb (S780) in M-S, MPF-R, T-S and TPF-R cells was performed using ImageJ software and normalized to GAPDH. Data are shown as area under the curve (AUC) normalized to loading control.

Supplementary Figure S8

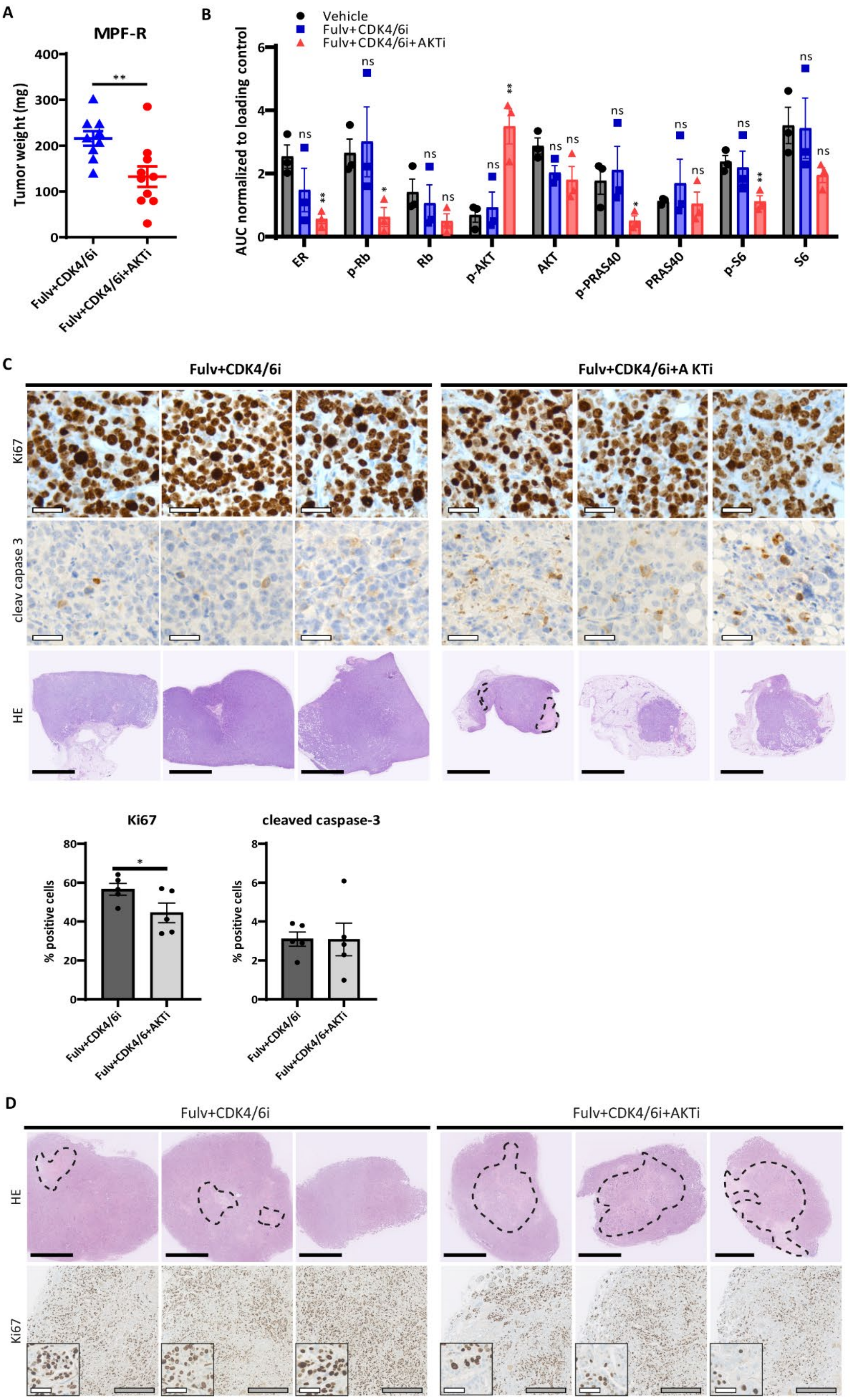

Fig. S8. Combined inhibition of ER, CDK4/6 and AKT suppresses tumor growth in xenograft models resistant to combined CDK4/6i and fulvestrant. (**A**) Evaluation of the efficacy of CDK4/6 inhibitor (CDK4/6i) palbociclib (50 mg/Kg bodyweight) combined with fulvestrant (Fulv, 100mg/Kg bodyweight; $N = 9$) or in triple combination with AKT inhibitor (AKTi) capivasertib (100mg/Kg bodyweight; $N = 10$) on MPF-R tumors weight at endpoint (week 7). CDK4/6i and AKTi were administered by oral gavage once daily for 5 days a week, while fulvestrant was administered subcutaneously once a week. Data are shown as mean endpoint tumor weight ± SEM. Asterisks indicate significant difference in two-tailed t-tests at the endpoint ($p < 0.05$). (**B**) Densitometry analysis of Western blot bands of key signal transduction proteins in 3 tumors of each group was performed using ImageJ software and normalized to GAPDH. Data are shown as mean area under the curve (AUC) normalized to loading control ± SEM. Asterisks indicate significant differences in two-tailed t-test ($p < 0.05$). (**C**) Representative micrographs of MPF-R tumor sections from 3 mice of each treatment group showing Ki67 and cleaved caspase-3 expression and HE by immunohistochemistry (scale bars, white: 100 µM, black: 2.5 mm). Quantification of Ki67 and cleaved caspase-3 was performed by ImageJ. Data are shown as mean ± SEM. Asterisks indicate significant differences by one-tailed t-test ($p < 0.05$). (**D**) Representative micrographs of immunohistochemical stainings of sections of the PDX KCC_P_3837-FPR resistant to combined palbociclib and fulvestrant treated with double or triple combination (3 mice of each treatment group) showing Ki67 expression and morphology by HE staining (scale bars, white: 100 µm, black: 2.5 mm, gray: 1 mm).

Supplementary Figure S9

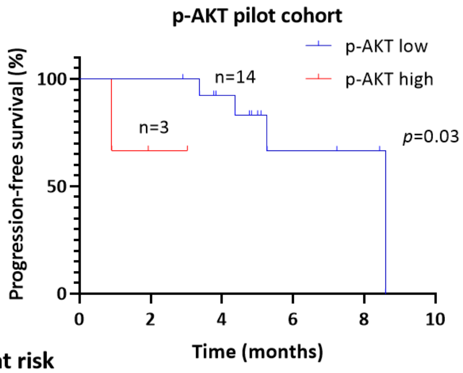

No. at risk						
p-AKT low	14	14	11	4	3	0
p-AKT high	3	2	0	0	0	0

Fig. S9. Evaluation of the correlation between p-AKT levels and progression-free survival (PFS) in a pilot cohort of ER+ metastatic breast cancer patients treated with combined CDK4/6i and endocrine therapy. Kaplan-Meier plots evaluating progression-free survival (PFS) according to p-AKT (S473) levels in ER+ metastatic lesions from a pilot cohort of ER+ breast cancer patients treated with endocrine therapy and CDK4/6i combination in the advanced setting. The cut-off value for high (H-score ≥ 150) vs. low (H-score < 150) was determined based on the survival significance. A two-sided *p* value ($p < 0.05$) was calculated using log-rank testing.

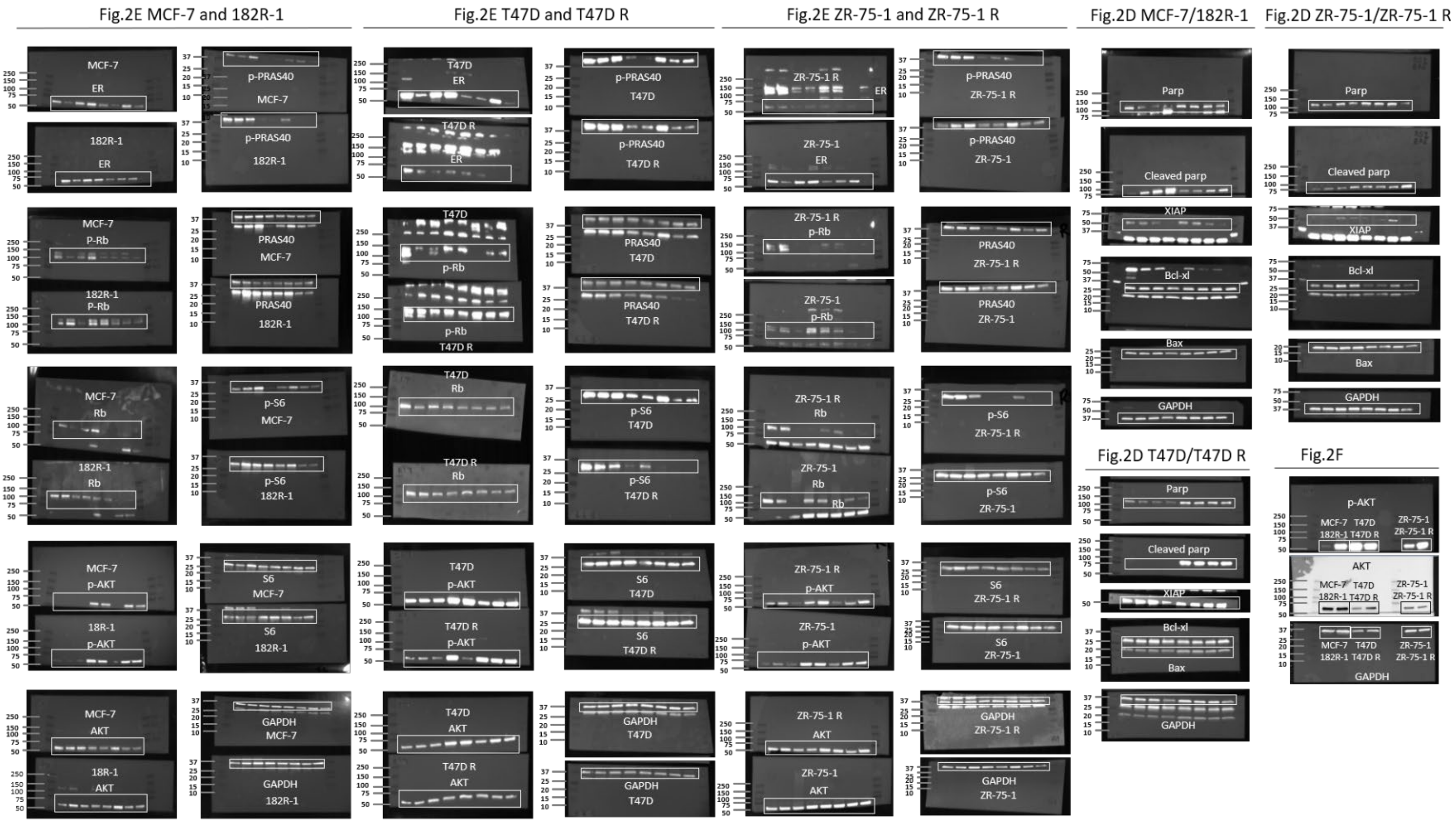

Fig. S10. Uncropped images of all Western blots shown in Fig. 2. Uncropped full scans of Western blots from the corresponding cropped Western blots shown in Fig. 2. Figure subpanel and molecular markers are indicated.

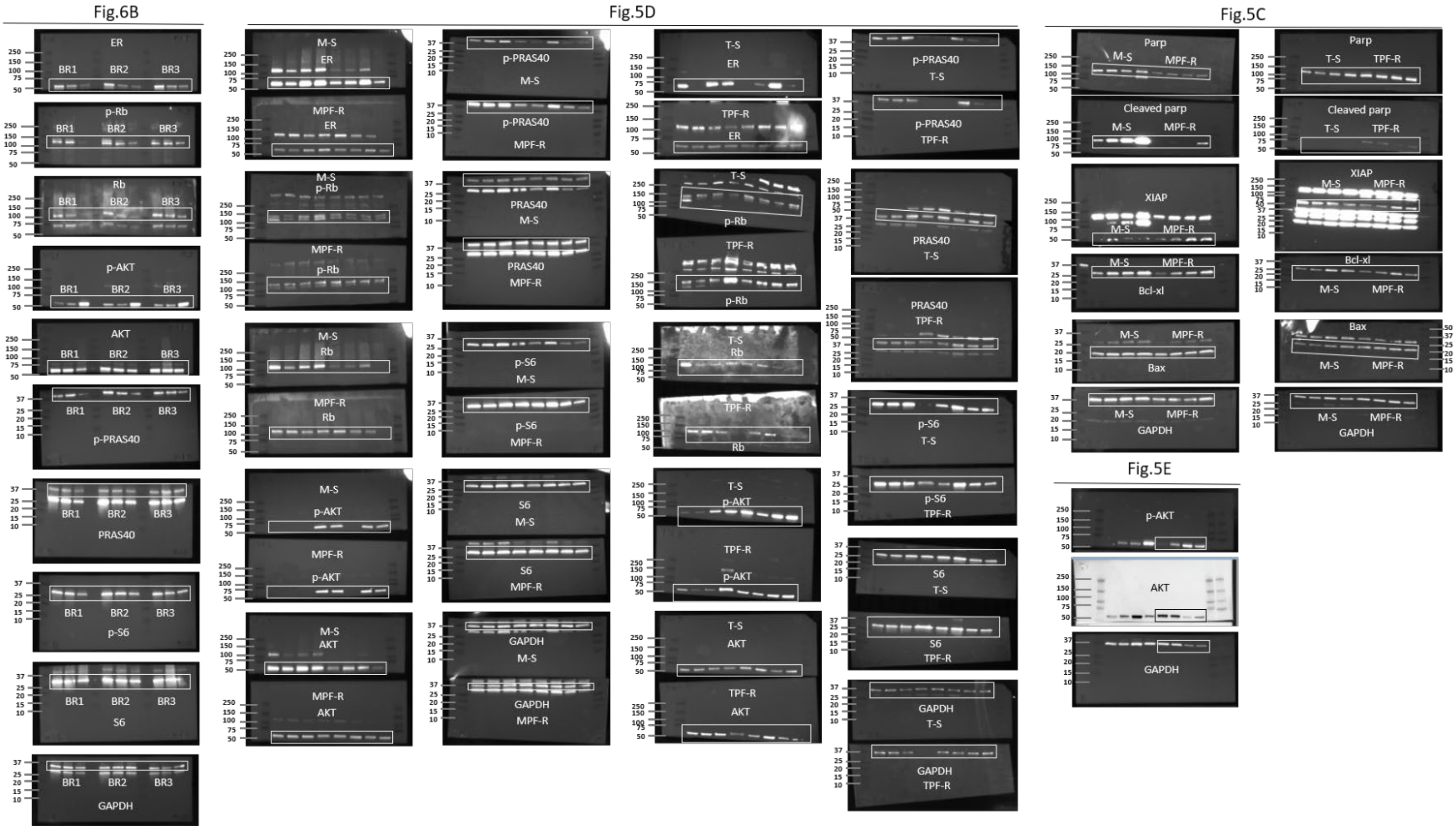

Fig. S11. Uncropped images of all Western blots shown in Figs. 5 and 6. Uncropped full scans of Western blots from the corresponding cropped Western blots shown in Figs. 5 and 6. Figure subpanel and molecular markers are indicated.

Table S1. Clinical and pathological characteristics of the primary tumor of ER+ breast cancer patients with advanced disease treated with CDK4/6i and endocrine treatment from pilot and validation cohorts according to p-AKT levels

	**Pilot**				**Validation**				**Pilot vs. validation**			
**Parameters**	**p-AKT low**	**p-AKT high**	N	p^a	**p-AKT low**	**p-AKT high**	N	p^a	**pilot**	**validation**	N	p^a
**Age at primary tumor**												
≤50 years	3	1	4	>0.99	27	6	33	0.79	4	33	37	0.28
>50 years	11	2	13		40	11	51		13	51	64	
**Size (mm) primary tumor**												
≤20	10	0	10	0.006	25	10	35	0.009	10	35	45	0.40
20-50	1	3	4		28	0	28		4	28	32	
>50	1	0	1		8	2	10		1	10	11	
unknown	2	0	2		6	5	11		2	11	13	
**Lymph node status primary tumor**												
positive	9	1	10	0.51	40	9	49	>0.99	10	49	59	>0.99
negative	3	1	4		15	3	18		4	18	22	
unknown	2	1	3		12	5	17		3	17	20	
**Grade primary tumor**												
I	3	2	5	0.46	18	2	20	0.30	5	20	25	0.18
II	2	0	2		20	8	28		2	28	30	
III	5	1	6		15	4	19		6	19	25	
unknown	4	0	4		14	3	17		4	17	21	
**Total**	**14**	**3**	**17**		**67**	**17**	**84**		**17**	**84**	**101**	

a Two-sided $\chi 2$ or Fisher's exact test

Table S2. Cox regression analysis of progression-free survival according to p-AKT expression and clinicopathological characteristics of ER+ breast cancer patients with advanced disease treated with CDK4/6i and endocrine treatment

**Variable**	**Hazard ratio (95% CI)**	***p*** [a]
p-AKT	2.07 (1.00-4.29)	0.049
Age at starting CDK4/6i	0.48 (0.20-1.16)	0.101
Site of relapse	1.23 (0.79-1.89)	0.360
No. metastatic sites	1.29 (0.88-1.88)	0.187
Chemotherapy	1.31 (0.71-2.41)	0.383
Time to recurrence	0.90 (0.64-1.29)	0.577
Line of therapy	3.05 (1.61-5.79)	0.001

[a] Cox proportional hazards model

Table S3. Distribution of the metastatic parameters included in the cox regression analysis between p-AKT-low vs. -high groups

Parameters	N (%) in p-AKT low	N (%) in p-AKT high
**Age at starting CDK4/6i**		
≤50 years	5 (7.5%)	2 (11.8%)
>50 years	62 (92.5%)	15 (88.2%)
**Site of relapse**		
Soft tissue	20 (29.9%)	5 (29.4%)
Bone	34 (50.7%)	5 (29.4%)
Visceral	13 (19.4%)	7 (41.2%)
**No. metastatic sites**		
1	18 (26.9%)	6 (35.3%)
2	19 (28.3%)	3 (17.6%)
≥ 3	30 (44.8%)	8 (47.1%)
**Chemotherapy metastatic setting**		
No	51 (76.1%)	11 (64.7%)
Yes	16 (23.9%)	6 (35.3%)
**Time of recurrence**		
<5 years	28 (41.8%)	5 (29.4%)
5-10 years	16 (23.9%)	7 (41.2%)
≥10 years	23 (34.3%)	5 (29.4%)
**Line of therapy CDK4/6i**		
1st	33 (49.3%)	6 (35.3%)
2nd or more	34 (50.7%)	11 (64.7%)
**Total**	**67**	**17**
