# Supplementary material for: Co-targeting CDK4/6 and AKT with endocrine therapy prevents progression in CDK4/6 inhibitor and endocrine therapy-resistant breast cancer
Source: Nat Commun. 2021 Aug 25;12:5112. doi: 10.1038/s41467-021-25422-9 (PMC8387387; doi:10.1038/s41467-021-25422-9)
Supplement: Supplementary file 3 — Reporting Summary [file 41467_2021_25422_MOESM3_ESM.pdf]

nature research

Corresponding author(s): Carla Alves

Last updated by author(s): Jul 23, 2021

Reporting Summary

Nature Research wishes to improve the reproducibility of the work that we publish. This form provides structure for consistency and transparency in reporting. For further information on Nature Research policies, see Authors & Referees and the Editorial Policy Checklist .

Statistics

For all statistical analyses, confirm that the following items are present in the figure legend, table legend, main text, or Methods section.

n/a	Confirmed	
☐	☒	The exact sample size (*n*) for each experimental group/condition, given as a discrete number and unit of measurement
☐	☒	A statement on whether measurements were taken from distinct samples or whether the same sample was measured repeatedly
☐	☒	The statistical test(s) used AND whether they are one- or two-sided<br>*Only common tests should be described solely by name; describe more complex techniques in the Methods section.*
☐	☒	A description of all covariates tested
☐	☒	A description of any assumptions or corrections, such as tests of normality and adjustment for multiple comparisons
☐	☒	A full description of the statistical parameters including central tendency (e.g. means) or other basic estimates (e.g. regression coefficient) AND variation (e.g. standard deviation) or associated estimates of uncertainty (e.g. confidence intervals)
☐	☒	For null hypothesis testing, the test statistic (e.g. *F*, *t*, *r*) with confidence intervals, effect sizes, degrees of freedom and *P* value noted<br>*Give P values as exact values whenever suitable.*
☒	☐	For Bayesian analysis, information on the choice of priors and Markov chain Monte Carlo settings
☐	☒	For hierarchical and complex designs, identification of the appropriate level for tests and full reporting of outcomes
☒	☐	Estimates of effect sizes (e.g. Cohen's *d*, Pearson's *r*), indicating how they were calculated

Our web collection on statistics for biologists contains articles on many of the points above.

Software and code

Policy information about availability of computer code

Data collection	No software was used.
Data analysis	STATA v16.0 (STATACorp), GraphPad Pism v8 (GraphPad Software, Inc.), ImageJ Java v8 (NIH), NDP.view v2 (Hamamatsu)

For manuscripts utilizing custom algorithms or software that are central to the research but not yet described in published literature, software must be made available to editors/reviewers. We strongly encourage code deposition in a community repository (e.g. GitHub). See the Nature Research guidelines for submitting code & software for further information.

Data

Policy information about availability of data

All manuscripts must include a data availability statement. This statement should provide the following information, where applicable:

- Accession codes, unique identifiers, or web links for publicly available datasets
- A list of figures that have associated raw data
- A description of any restrictions on data availability

Survival analyses and immunohistochemistry data, are not publicly available to protect patient privacy, but will be made available to authorized researchers who have an approved Institutional Review Board application and have obtained approval from The Regional Committees on Health Research Ethics for Southern Denmark. Please contact the corresponding author with data access requests. All other datasets generated during the study will be made available upon request from the corresponding author, Henrik Ditzel, email address: hditzel@health.sdu.dk. Uncropped Western blots are part of the supplementary files.The authors declare that the data supporting the findings of this study are available within the paper and its supplementary information files. Source data are provided with this paper.

Field-specific reporting

Please select the one below that is the best fit for your research. If you are not sure, read the appropriate sections before making your selection.

- [x] Life sciences
- [ ] Behavioural & social sciences
- [ ] Ecological, evolutionary & environmental sciences

For a reference copy of the document with all sections, see nature.com/documents/nr-reporting-summary-flat.pdf

Life sciences study design

All studies must disclose on these points even when the disclosure is negative.

Sample size

Power calculations were performed to determine that the number of animals and tumor samples should be sufficient to determine significant differences between the different groups.

Data exclusions

No data was excluded.

Replication

All experiments were performed at least twice, but more often three times. All atempts at replication were successful.

Randomization

When tumor xenografts reached 50 mm3 or 100-150 mm3 (depending on the experiment), the mice were randomized into treatment groups based on equal tumor sizes. PDXs: Two weeks after implantation, mice were randomized to two treatment arms based on equal tumor sizes. For the clinical samples, patients with metastatic biopsies from 2019 (N = 17) were included in a pilot cohort that was used to select the cut-off based on the survival significance. Patients with metastatic biopsies obtained before 2019 (N = 84) were included in the validation cohort and the cut-off selected in the pilot cohort was applied to stratify patients into p-AKT low and high groups. No randomization was required for in vitro studies.

Blinding

Evaluation of the immunohistochemistry staining was performed by a skilled breast pathologist in a blinded setup. Evaluation of xenograft and PDX tumors volume was not blinded as the same person performed the administration of drugs and evaluated tumor growth. For the metastatsis model, the amount of metastasis (defined, approximately, as > 2500 µm2) relative to lung area was determined by ImageJ analysis in a blinded setup. No blinding was required for the in vitro studies.

Reporting for specific materials, systems and methods

We require information from authors about some types of materials, experimental systems and methods used in many studies. Here, indicate whether each material, system or method listed is relevant to your study. If you are not sure if a list item applies to your research, read the appropriate section before selecting a response.

Materials & experimental systems

n/a	Involved in the study	
[ ]	[x]	Antibodies
[ ]	[x]	Eukaryotic cell lines
[x]	[ ]	Palaeontology
[ ]	[x]	Animals and other organisms
[ ]	[x]	Human research participants
[x]	[ ]	Clinical data

Methods

n/a	Involved in the study	
[x]	[ ]	ChIP-seq
[x]	[ ]	Flow cytometry
[x]	[ ]	MRI-based neuroimaging

Antibodies

Antibodies used

anti-ER (Thermo Scientific, #MA5-14501, clone SP1), anti-GAPDH (Santa Cruz biotechnology, #sc-32233, clone 6C5), anti-p-Rb S780 (Cell Signaling, #3590, clone C84F6), anti-Rb (Cell Signaling, #9309, clone 4H1), anti-p-AKT S473 (Cell Signaling, #4060, clone D9E), anti-AKT (pan) (Cell Signaling, #4685, clone 11E7), anti-p-PRAS40 T246 (Cell Signaling, #2997, clone C77D7), anti-PRAS40 (Cell Signaling, #2691, clone D23C7), anti-p-S6 S235/236 (Cell Signaling, #2211, polyclonal), anti-S6 (Cell Signaling, #2217, clone 5G10), anti-cleaved PARP (Cell Signaling, #9541, polyclonal), anti-PARP (Cell Signaling, #9532, clone 46D11), anti-Xiap (Cell Signaling, #2042, polyclonal), anti-Bcl-xl (Cell Siganling, #2762, polyclonal), anti-Bax (Cell Signaling, #2772, polyclonal) , horseradish peroxidase (HRP)-conjugated goat anti-mouse (Daki, #P0447, polyclonal), HRP-conjugated goat anti-rabbit (Dako, #P0448, polyclonal), Ki67 (790-4286, Ventana Medical Systems) , anti-cleaved caspase-3 (9664, Cell Signaling Technology), anti-cytokeratin antibody (M351501-2, Agilent).

Validation

All antibodies are commercially available and have been validated by the company for Western blotting (anti-ER, anti-GAPDH, anti-p-Rb S780, anti-Rb, anti-p-AKT S473, anti-AKT (pan), anti-p-PRAS40 T246, anti-PRAS40, anti-p-S6 S235/236, anti-S6, anti-cleaved PARP, anti-PARP, anti-Xiap, anti-Bcl-xl, anti-Bax) or immunohistochemistry (Ki67, anti-cleaved caspase-3, anti-cytokeratin, anti-p-AKT S473.

Eukaryotic cell lines

Policy information about cell lines

Cell line source(s)	The original MCF-7and T47D cell lines were obtained from the Breast Cancer Task Force Cell Culture Bank, Mason Research Institute. The original ZR-75-1 cell line was obtained from the American Type Culture Collection (ATCC)
Authentication	All cell lines were authenticated by Short Tandem Repeat (STR) DNA profiling.
Mycoplasma contamination	All cell lines were tested negative for mycoplasma contamination using Lonza MycoAlert Kit.
Commonly misidentified lines (See ICLAC register)	No commonly misidentified lines were used in this study.

Animals and other organisms

Policy information about studies involving animals; ARRIVE guidelines recommended for reporting animal research

Laboratory animals	For the xenografts, the recipients were 7-week-old female NOG CIEA mice, which were purchased from Taconic. For the PDXs, 6–8-week-old female NOD-SCID-IL2γR−/− mice (Australian BioResources Pty Ltd).
Wild animals	No wild animals were used in this study.
Field-collected samples	No field-collected samples were used in this study.
Ethics oversight	For the xenografts, experiments were approved by the Experimental Animal Committee of The Danish Ministry of Justice and were performed at the animal core facility at University of Southern Denmark. For the PDXs, experiments were approved by the Garvan Institute of Medical Research Animal Ethics Committee (protocols 15/25, 18/20 and 18/26).

Note that full information on the approval of the study protocol must also be provided in the manuscript.

Human research participants

Policy information about studies involving human research participants

Population characteristics	ER+ breast cancer patients treated with combined CDK4/6i and endocrine therapy in the advanced setting were selected retrospectively by database extraction from the archives of the Department of Pathology at Odense University Hospital (OUH) (N = 115). The inclusion criteria were ER+ breast cancer patients treated with combined CDK4/6i and endocrine therapy in the advanced setting who had undergone surgery or biopsy for advanced stage disease at OUH, and for whom complete clinical information and pathological verification that the metastatic lesion was of breast cancer origin was available. Exclusion criteria were insufficient tumor material in the FFPE block and metastatic biopsy only available after commencing treatment with combined CDK4/6i and endocrine therapy. These parameters yielded N = 101 patients. Patients with metastatic biopsies from 2019 (N = 17) were included in a pilot cohort that was used to select the cut-off based on the survival significance. Patients with metastatic biopsies obtained before 2019 (N = 84) were included in the validation cohort and the cut-off selected in the pilot cohort was applied to stratify patients into p-AKT low and high groups. Tumors were defined ER+ if ≥ 1% of the tumor cells were stained positive. Progression-free survival (PFS) was defined as the time from initiation of combined endocrine therapy and CDK4/6i treatment until disease progression or death. Age, number of metastasis, time to recurrence, line of therapy and site of relapse were considered relevant covariates of the metastatic disease and included in the cox regression model.
Recruitment	Retrospective database extraction from the archives of the Department of Pathology at Odense University Hospital (OUH) (N = 115). The inclusion criteria: ER+ breast cancer patients treated with combined CDK4/6i and endocrine therapy in the advanced setting who had undergone surgery or biopsy for advanced stage disease at OUH, and for whom complete clinical information and pathological verification that the metastatic lesion was of breast cancer origin was available. Exclusion criteria: insufficient tumor material in the FFPE block and metastatic biopsy only available after commencing treatment with combined CDK4/6i and endocrine therapy. These parameters yielded N = 101 patients. Patients with metastatic biopsies from 2019 (N = 17) were included in a pilot cohort that was used to select the cut-off based on the survival significance. Patients with metastatic biopsies obtained before 2019 (N = 84) were included in the validation cohort and the cut-off selected in the pilot cohort was applied to stratify patients into p-AKT low and high groups. No self-selection or other biases were present.
Ethics oversight	All clinical samples were coded to maintain patient confidentiality and studies were approved by the Ethics Committee of the Region of Southern Denmark and Copenhagen and Frederiksberg Counties (approval no S-2008-0115).

Note that full information on the approval of the study protocol must also be provided in the manuscript.

Clinical data

Policy information about clinical studies

All manuscripts should comply with the ICMJEguidelines for publication of clinical research and a completedCONSORT checklist must be included with all submissions.

Clinical trial registration	N/A

Study protocol	N/A
Data collection	N/A
Outcomes	N/A
